# Melatonin Ameliorates Inflammation and Oxidative Stress by Suppressing the p38MAPK Signaling Pathway in LPS-Induced Sheep Orchitis

**DOI:** 10.3390/antiox9121277

**Published:** 2020-12-14

**Authors:** Shou-Long Deng, Bao-Lu Zhang, Russel J. Reiter, Yi-Xun Liu

**Affiliations:** 1 CAS Key Laboratory of Genome Sciences and Information, Beijing Institute of Genomics, Chinese Academy of Sciences, Beijing 100101, China; dengsl@big.ac.cn; 2 Marine Consulting Center of Natural Resources of the People’s Republic of China, Beijing 100071, China; zhangbaolu80@163.com; 3 Department of Cell Systems and Anatomy, UT Health San Antonio, San Antonio, TX 78229, USA; 4 State Key Laboratory of Stem Cell and Reproductive Biology, Institute of Zoology, Chinese Academy of Sciences, Beijing 100101, China

**Keywords:** melatonin, TLR4, testicular macrophages, p38MAPK, sheep

## Abstract

Gram-negative bacterial infections of the testis can lead to infectious orchitis, which negatively influences steroid hormone synthesis and spermatogenesis. Lipopolysaccharide (LPS), a major component of the Gram-negative bacterial cell wall, acts via toll like receptors 4 (TLR4) to trigger innate immune responses and activate nuclear factor kappa B signaling. The protective mechanisms of melatonin on LPS-induced infectious orchitis have not been reported. Herein, we developed an LPS-induced sheep infectious orchitis model. In this model, the phagocytic activity of testicular macrophages (TM) was enhanced after melatonin treatment. Moreover, we found that melatonin suppressed secretion of TM pro-inflammatory factors by suppressing the p38MAPK pathway and promoting Leydig cell testosterone secretion. Expressions of GTP cyclohydrolase-I and NADPH oxidase-2 were reduced by melatonin while heme oxygenase-1 expression was up-regulated. Thus, melatonin reduced the severity of LPS-induced orchitis by stimulating antioxidant activity. The results of this study provide a reference for the treatment of acute infectious orchitis.

## 1. Introduction

Toll like receptor 4 (TLR4) is an innate immune pattern recognition receptor, which recognizes lipopolysaccharide (LPS), a major component of the cell wall of Gram-negative bacteria. Recently, it was predicted that this receptor may also recognize molecular patterns of SARS-CoV-2 [1]. TLR4 activates nuclear factor kappa B (NF-кB) pathway, mediates inflammatory responses and initiates oxidative stress, making it a potential target for the treatment for many acute and chronic diseases. RNA virus, genital tract bacterial infection and tissue damage are the main reasons for orchitis. Brucella, a Gram-negative bacterium, causes zoonotic diseases such as orchitis. During the acute phase of infection, Brucella produces male infertility by disrupting reproductive hormones and interrupting spermatogenesis. When infection occurs, LPS induces testicular macrophages (TM) to produce inflammatory factors and free radicals, which inhibit gonadal steroidogenesis including suppression of testosterone (T), a key molecule in maintaining normal spermatogenesis [2]. TM are essential for the maintenance of testicular immune privilege and display reduced pro-inflammatory capacity [3]. In patients with infertility-related orchitis, the number of macrophages with strong phagocytic activity in testicular interstitial tissue is decreased; conversely, the number of macrophages that secrete abundant inflammatory cytokines is increased. TM responds to TLR4 activation with p38 mitogen-activated protein kinase (MAPK) and NF-κB followed by the release of inflammatory agents and the accumulation of free radical-mediated oxidative damage in experimental autoimmune orchitis [4].

Oxidative damage induced by TM causes Leydig cell dysfunction, perturbed mitochondrial physiology, the inability of steroidogenic acute regulatory protein (StAR) to promote cholesterol transport into mitochondria, and inhibition of steroid hormone production [5]. The production of nitric oxide (NO) is likewise changed. Guanosine triphosphate cyclohydrolase I (GCHI) is a rate-limiting enzyme for tetrahydrobiopterin synthesis, which is necessary for the activation of inducible nitric oxide synthetase (iNOS). The transcription of iNOS is regulated by the NF-𝜅B pathways [6]. Reactive oxygen species (ROS) are mainly generated by nicotinamide adenine dinucleotide phosphate (NADPH) oxidase. The activity of NF-κB is regulated by the direct intervention of TLR4 with NADPH oxidase 2 (Nox2), subunits of NAPDH oxidase [7].

Melatonin, a secretory product of the pineal gland, penetrates the blood-testis barrier (BTB) and has a variety of physiological actions including functioning as an antioxidant, regulating reproductive hormone secretion and influencing the immune system [8,9,10,11]. Melatonin up-regulates the expression of GATA-4 transcription factors and promotes the secretion of testosterone by sheep Leydig cells [12]. Melatonin at physiological concentrations increases the phagocytic index of TM [13]. In patients with infertility, melatonin shows anti-inflammatory effects on TM. Melatonin limits NF-𝜅B transport into the nucleus and reduces the level of pro-inflammatory factors [14,15]. In vitro, the addition of melatonin reduces p38MAPK phosphorylation and down-regulates the inflammatory response in LPS-stimulated mouse macrophages [16]. Orchitis disrupts the balance of oxidation/anti-oxidant and inflammation/anti-inflammation networks. At present, the anti-inflammatory treatment of orchitis is not adequate. This current study explores the intervention mechanism of melatonin in inflammatory response and oxidative stress in LPS-induced infectious orchitis.

## 2. Materials and Methods

### 2.1. Ethics Statement

Surgical biopsies of sheep were performed at the experimental farm of the China Agricultural University; the procedure was carried out by strictly following the protocol approved by the Animal Welfare Committee of the China Agricultural University (Permit Number: XK662).

### 2.2. Chemicals and Reagents

All chemical agents used in this study were obtained from Sigma-Aldrich Chemical Company (St. Louis, MO, USA).

### 2.3. Isolation and Culture of Testicular Macrophages

Testicular tissues were collected at an abattoir. The materials were placed in physiological saline at 4 °C and transported to the laboratory within 2 h. The testicular tissues were washed three times with PBS supplemented with 100 IU/mL penicillin and 100 mg/mL streptomycin. Testicular macrophages were isolated and cultured following a previously published method [17,18]. After removing tunica albuginea, the seminiferous tubules were collected, placed in PBS containing 0.03% collagenase I and incubated at 4 °C for 40 min. Samples were removed with a pipette and then gently separated with tweezers, keeping the seminiferous tubules intact. The cell suspension was filtered using a 40-mesh sieve; the material recovered contained mainly Leydig cells and TM. Cell suspension were removed by centrifugation (1000 rpm/min, 5 min), and cells were re-suspended in RPMI-1640 (Gibco, Grand Island, NY, USA) medium including 10% fetal bovine serum and then placed in a culture flask. Cells were maintained in 5% CO_2_, 34 °C incubators for 30 min. The non-adherent cells and supplemental medium were removed. The adherent cells were transferred to a flask and refrigerated at 4 °C. When cell contraction was observed, medium was pipetted to detach the cells. Cell concentration was adjusted to 1 × 10^5^/mL, and re-inoculated on 6-well plates. Purification was estimated by immunofluorescence, TMs were identified using a cell surface marker CD11b.

The media were replaced 24 h later with serum-free RPMI-1640 for another 24 h. To activate macrophages, 100 ng/mL LPS were introduced into the medium. In the cultured cells, the medium melatonin (10^−7^ M) was added in serum-free RPMI with either SB203580 (10^−6^ M) (p38MAPK inhibitor), the NF-κB pathway inhibitor PDTC (10^−6^ M), Nrf2 inhibitor ML385 (10^−6^ M), GCH1 inhibitor DAHP (10^−6^ M), or the Nox2 inhibitor GSK2795039 (10^−6^ M), respectively. Cells and culture media were collected after 6 h of culture. MTT (Amresco, Solon, OH, USA) was employed to detect phagocytosis of testicular macrophages according to a previously published method [19]. Flow cytometry was used to measure expression of CD163 (Cell Signaling Technology Inc., Beverly, MA, USA) and intracellular ROS (Genmed Scientifics, Inc., Wilmington, DE, USA) in macrophages.

### 2.4. Leydig Cells Co-Culture with Testicular Macrophages

Isolation of Leydig cells for culturing was preformed according to a procedure described in [20]. The co-culture experiment of testicular macrophages and Leydig cells has also been previously described [21,22]. In the co-culture system, testicular macrophages (0.4 × 10^5^/mL) were cultured in a transwell plate; Leydig cells (1.6 × 10^5^/mL) were cultured in the bottom of the plate (Corning Company, 3491, New York, NY, USA). The medium without or with melatonin (10^−7^ M), 100 ng/mL LPS and polymixin B (10^−6^ M) (TLR4 inhibitor) (InvivoGen, San Diego, CA, USA) was added to the Leydig cell culture group and co-culture group, respectively. Each preparation was cultured at CO_2_ 5%, 34 °C. The cells and medium were collected 6h later for further analysis.

### 2.5. Effects of Regionally Injecting LPS with or without Melatonin into Testes

Twelve adult sheep were randomly divided into four groups and LPS was injected as LPS alone or in combination with melatonin into different regions of each testis [23]. Group A, sheep (*n* = 3) were injected with 50 µL physiological saline and these testes served as the controls; Group B animals received an intra-testicular injection of LPS 10 µg in 50 µL of saline (*n* = 3). Group C sheep received 10 µg/50 µL LPS followed 30 min later with an intra-testicular injection of melatonin (10 µg/50 µL saline (*n* = 3). Group D was injected with melatonin only (10 µg/50 µL saline) (*n* = 3). Six hours after the injections, the damaged areas of the testes were surgically removed with the sampling site being closed by suturing. A portion of the surgically-removed tissue was fixed with paraformaldehyde. Paraffin sections were prepared. Hematoxylin-eosin (HE) staining was used to observe the pathological changes. CD11b (Santa Cruz Biotechnology, Santa Cruz, CA, USA) and CD163 expressions were observed by immunohistochemistry. RNA, protein and testicular single cell suspensions were prepared with the remaining part of the sample. Testicular cells were stained with PI. Ploidy of cell and ROS levels were analyzed by flow cytometry. 

### 2.6. Quantitative Reverse Transcription—(qRT-) PCR

Total protein and RNA were extracted from other portions of the testicular tissue, TMs and Leydig cells were analyzed using a RNA/protein extraction kit (Tiangen Biotechnology, Beijing, China) in accordance with the protocols. cDNA was obtained by reverse transcription. Subsequently, mRNA transcripts of CD11b and Nox2 in testicular tissue were detected by qRT-PCR. Transcription of Nox2 in cultured testicular macrophages was quantitatively measured. Expressions of 3β-hydroxysteroid dehydrogenase (3β-HSD), steroidogenic factor 1 (SF1) and StAR in cultured Leydig cells were detected; β-actin was used as an internal reference. Primer sequences were as shown: CD11b (EF206308.1) F-CGC TGCT GGC CTG GCC, R-TCG GAT GAA GAA GTT TGT CT; Nox2 (EF373654.1) F-TCC ATC CGC ATC GTG, R-GTT AGT GGG AGC AGG GAT C; 3β-HSD (FJ007375.1) F-ATC CAC ACC AGC ACC ATA G, R-TAC ACT TGT GCC TTG AGG; SF1 (XM013967971.1) F-TAC CTC TAC CCT GCC TTC CCT, R-CCG CAC TTG GTC CTC ATC A; StAR (XM013975437.1) F-GCA GAA GGG TGT CAT CAG AGG C, R-GGC AAA ATC CAC TTG GGT CT; β-actin (AF481159.1) F-CAC GGT GCC CAT CTA CGA G, R-CCT TGA TGT CAC GGA CGA TTT. The qRT-PCR reaction was performed using a Real-Time Master Mix SYBR Green kit (Tiangen, Beijing, China) and a MX300P system (Stratagene, San Diego, CA, USA). Fold changes in the gene expression were calculated using the 2*^−ddct^* method.

### 2.7. Western Blots

The proteins were electrophoresed under reducing conditions in 12% SDS-PAGE gels and transferred to nitrocellulose membranes. Bovine serum albumin of 5% (*w/v*) was used to block the membranes and then incubated at 4 °C for 12 h with primary antibodies against TLR4 (Abcam, Cambridge, UK), androgen receptor (AR) (Abcam), p-p38MAPK/p38MAPK (Santa Cruz Biotechnology), p-Nrf2/Nrf2 (Santa Cruz Biotechnology), p-NF-𝜅B/NF-𝜅B (Abcam), p-c-Jun N-terminal kinase (JNK)/JUK (Santa Cruz Biotechnology), or p-extracellular regulated protein kinase (ERK)1/2/ERK1/2 (Santa Cruz Biotechnology). β-tubulin served as a control. Then, the samples were incubated with enzyme-labeled secondary antibodies corresponding to the species of primary antibody at room temperature for 1 h. Optical densities were quantified by scanning densitometry and expressed in arbitrary units determined by Image J software 1.8.0 (National Institute of Health, Bethesda, MD, USA).

### 2.8. ELISA

Inflammatory agents including tumor necrosis factor alpha (TNF-α), interleukin (IL)-1β, IL-6 and IL-10, TLR4, T, GCH1, heme oxygenase-1 (HO-1), and melatonin were detected in testicular tissue suspensions and cultured cell suspensions with ELISA (Hermes Criteria Biotechnology, Vancouver, BC, Canada). Furthermore, the monocyte chemoattractant protein-1 (MCP-1) content was also evaluated in cell suspensions. All experimental procedures were performed according to the kit instructions.

### 2.9. Detection of Metabolites and Proteins Associated with Oxidative Stress

NO (A013-1-1) and malondialdehyde (MDA) (A003-1-2) content as well as iNOS (A014-1-2), NADPH oxidase (A127-1-1), cyclooxygenase-2 (COX-2) (H200), and the total antioxidant capacity (TAC) (A015-1-1) in the testicular tissue and cultured cell suspension were estimated. Furthermore, superoxide dismutase (SOD) (A001-1-2), catalase (CAT) (A007-1-1), and glutathione (GSH) (A006-1-1) were also detected in cultured testicular macrophages. The detection method was by spectrophotometry in accordance with the manual supplied with the detection kit (Nanjing Jiancheng Bioengineering Institute, Nanjing, China) [8].

### 2.10. Statistical Analysis

All experiments were repeated at least three times. One-way ANOVA was used to determine statistical significance followed by the Duncan’s test to determine the statistical significance between the relative groups (SAS Institute, Cary, NC, USA). All data were expressed as mean ± S.E.M. Differences were considered to be significant when *p* < 0.05.

## 3. Results

### 3.1. Low Testosterone Levels in LPS Contributes to Orchitis Tissues

**Figure 1:**
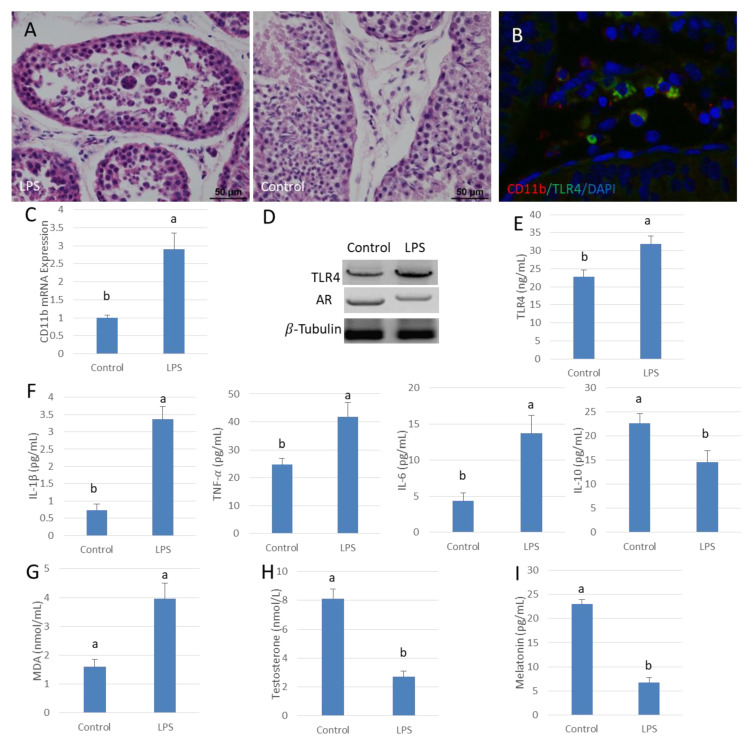
Lipopolysaccharide (LPS) reduces testosterone content in sheep testis. (**A**) Testis HE stains. Testes injected with saline were used as the control. Spermatogenesis was disturbed in the LPS injection group (left). (**B**) Testicular tissue immunofluorescence, CD11b (Red), TLR4 (Green) and DAPI (Blue). (**C**) CD11b gene mRNA expression in the LPS injection group. (**D**) Western blot analysis of TLR4 and AR in testis. (**E**,**F**) ELISA testing of TLR4, IL-1β, TNF-α, IL-6 and IL-10 protein expression. (**G**) MDA content in the LPS infected testis group. (**H**,**I**) ELISA detection of testosterone and melatonin concentrations. Data are shown as mean ± SEM. Different superscript letters (a, b) in each column represent statistically significant differences (*p* < 0.05).

A model of acute orchitis was established by injecting LPS into the testis of sheep. HE stained sections showed that spermatogenesis was disturbed by the LPS-induced infection. The spermatogenic epithelium was thin and vacuoles were present in many of the remaining cells. There were spermatogonia found in some seminiferous tubules but mature sperm were rare (Figure 1A). The infiltration of inflammatory cells was apparent between seminiferous tubules. The mRNA expression of macrophage marker gene CD11b was significantly higher in the LPS-treated testes than in the control organs (*p* < 0.05) (Figure 1B,C). Additionally, TLR4 protein expression increased significantly with increased expression of downstream pro-inflammatory cytokines, such as IL-6, TNF-𝛼 and IL-1β; these changes were accompanied by enhanced oxidative damage. The content of IL-10 was significantly lower than that of the control testes (*p* < 0.05) (Figure 1D–G). Translation of AR was decreased in testis along with significantly lower testosterone levels compared to those in the control organs (*p* < 0.05) (Figure 1D,H). In addition, the melatonin content in testis after LPS infection was significantly depressed compared to that of the control testes (*p* < 0.05) (Figure 1I).

### 3.2. Melatonin Enhances Phagocytosis of Testicular Macrophages

**Figure 2:**
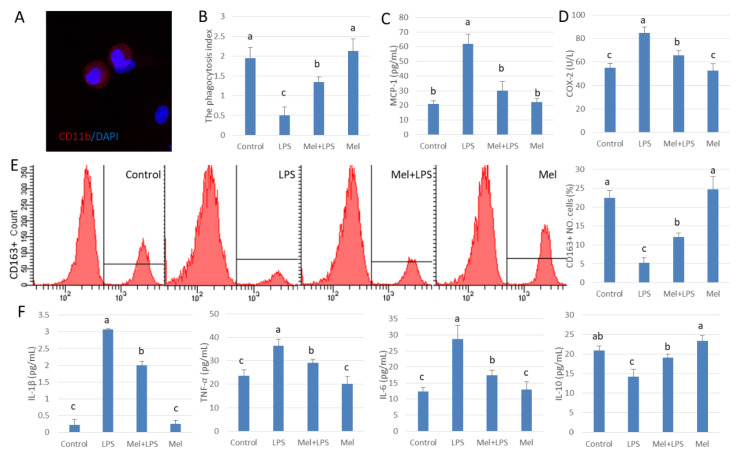
Melatonin improves phagocytosis in LPS-treated testicular macrophages. (**A**) Immunofluorescence stain in cultured testicular macrophages, CD11b (Red) and DAPI (Blue). (**B**) Phagocytosis. (**C**,**D**) ELISA was used to detect the expressions of MCP-1 and COX-2. (**E**) Expression of CD163 was analyzed by flow cytometry. (**F**) ELISA detection IL-1β, TNF-α, IL-6 and IL-10 protein expression. Experiments were divided into the control group, the LPS infection group, and the Mel+LPS and Mel group. Data are shown as mean ± SEM. Different superscript letters (a–c) in each column representative statistically significant differences (*p* < 0.05).

For further study, we isolated and cultured testicular macrophages in vitro (Figure 2A). We found that the index of phagocytosis in testicular macrophages decreased significantly in the LPS infected group (*p* < 0.05). Melatonin reduced phagocytosis by LPS-damaged testicular macrophages (Figure 2B) and lowered MCP-1 and COX-2 expression (Figure 2C,D). In testicular macrophages, CD163+ marked cells increased after melatonin treatment (Figure 2E), which indicates the number of phagocytes increased. Pro-inflammatory agents including IL-1β, TNF-𝛼 and IL-6 were elevated. After supplementation with melatonin, the levels of IL-1β, TNF-α, IL-6 decreased while that of IL-10 increased in the LPS infected group (*p* < 0.05) (Figure 2F), These results suggest that melatonin reduces the number of macrophages that secrete damaging cytokines and increase the number of phagocytic macrophages.

### 3.3. Melatonin Decreases TLR4-Mediated Inflammatory Genes via Inhibition of p38/MAPK Signaling Pathway

**Figure 3:**
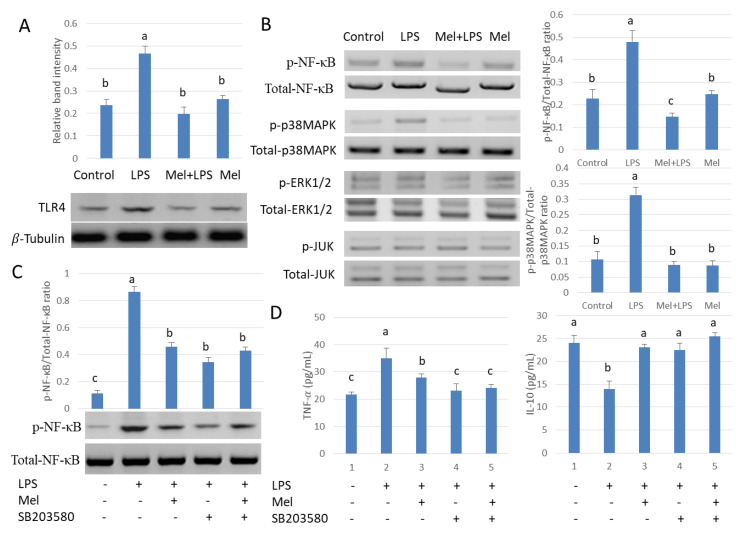
Melatonin regulates NF-𝜅B expression through the p38/MAPK signaling pathway in TM. Western blotting detection (**A**) expression of TLR4 and (**B**) p-p38MAPK/p38MAPK, p-JUN/JUN, p-ERK1/2/ERK1/2 and p-NF-кB/NF-кB. Four groups were used in this study: the control, LPS infection, Mel+LPS and Mel. (**C**) p-NF-𝜅B/NF-𝜅B expression Western Blotting detects. (**D**) TNF-𝛼 and IL-10 protein expressions detected by ELISA. TM treated with LPS, Mel, and/or SB203580. Data are shown as mean ± SEM. Different superscript letters (a–c) in each column representative statistically significant differences (*p* < 0.05).

To study the role of melatonin in inflammatory agent activation, TLR4 expression was examined. Results showed that LPS increased the expression of testicular macrophage TLR4; conversely, melatonin reduced this effect (Figure 3A). The phosphorylation state of p38MAPK, JUN, ERK1/2 and NF-𝜅B also was examined. Protein expression patterns of phosphorylated p38MAPK and NF-𝜅B were found to be the same as TLR4 (Figure 3B). Subsequently, we treated testicular macrophages with p38MAPK inhibitor SB203580 in vitro. The phosphorylated protein expression of NF-κB decreased after SB203580 was introduced in the culture system (Figure 3C). The downstream inflammatory factor TNF-𝛼 also decreased and the anti-inflammatory factor IL-10 protein content was elevated compared to the LPS-stimulated group (*p* < 0.05) (Figure 3D). The result shows melatonin decreased TLR4-mediated inflammatory genes via inhibition of the p38/MAPK signaling pathway in TM.

### 3.4. Melatonin Inhibits GCH1 and Nox2, and Decreased iNOS and NADPH Oxidase Activity in Testicular Macrophages

**Figure 4:**
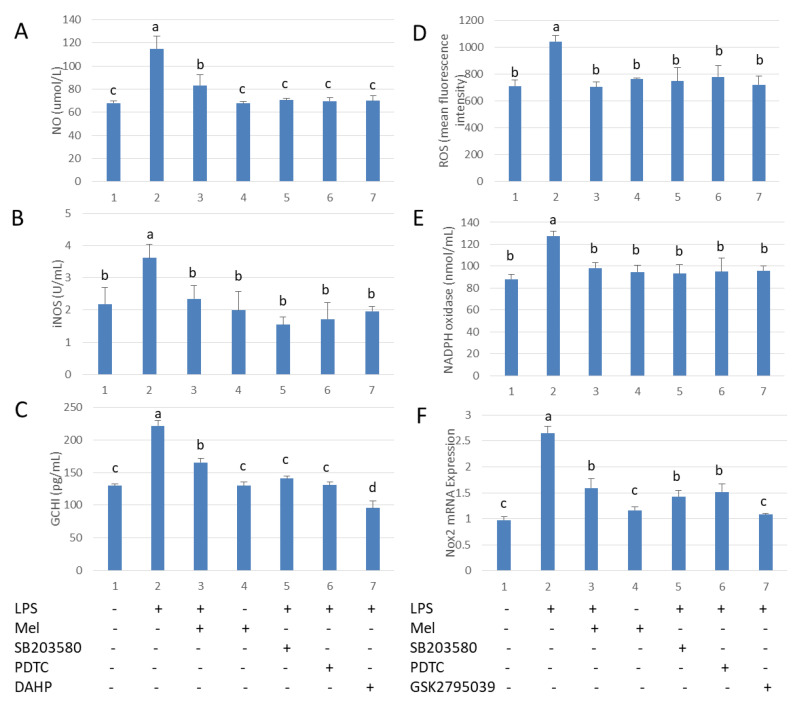
Melatonin reduces GCH1 activity and Nox2 mRNA expression in LPS-stimulated testicular macrophages. (**A**) NO content. (**B**) iNOS activation. (**C**) ELISA detection of GCH1 protein expression. TM treated with LPS, Mel, PDTC, DAHP and/or SB203580. (**D**) Flow cytometry analysis of ROS content. (**E**) NADPH oxidase activation. (**F**) mRNA expression of Nox2. TM treated with LPS, Mel, PDTC, GSK2795039 and/or SB203580. Data are shown as mean ± SEM. Different superscript letters (a–d) in each column representative statistically significant differences (*p* < 0.05).

We explored the mechanism by which melatonin reduces free radical damage in LPS stimulated-testicular macrophages. After challenge with LPS, the levels of NO and ROS were significantly increased. Melatonin treatment reduced these parameters. The effects of melatonin were prevented by adding SB203580 and PDTC. In addition, LPS up-regulated both GCH1 and Nox2 transcription. iNOS activity was found to be reduced after supplementation with DAHP, a GCH1 activation inhibitor (Figure 4A–C). Nox2 inhibitor (GSK2795039) lowered NADPH oxidase activation in LPS-stimulated TM (Figure 4D–F). These findings suggest LPS-stimulated macrophages produce NO and ROS via activation of TLR4. Melatonin significantly reduced the levels of NO and ROS by inhibiting the p38MAPK/GCH1/iNOS signaling pathway and the p38MAPK/Nox2/NADPH oxidase signaling pathway in TM.

### 3.5. Melatonin Attenuates LPS Oxidative Damage of Testicular Macrophages through Nrf2/HO-1 Pathways

**Figure 5:**
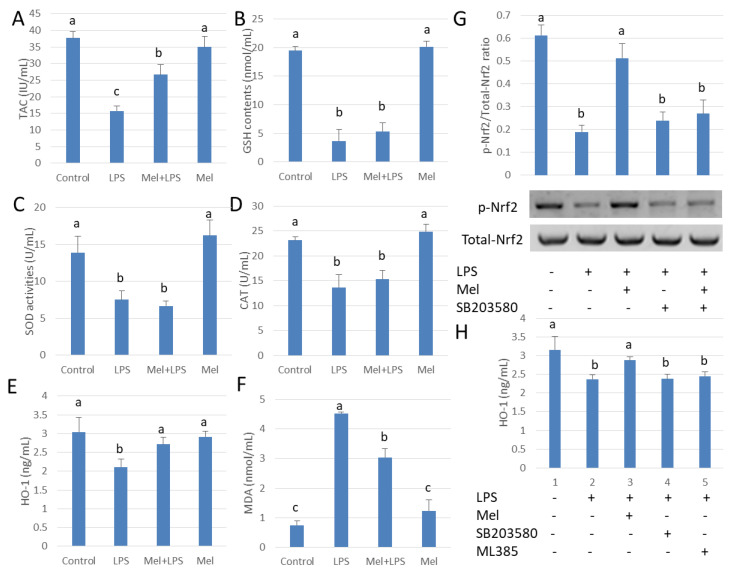
Melatonin up-regulated HO-1 expression attenuates LPS-induced oxidative damage in testicular macrophages. (**A**) TAC activation. (**B**) GSH activation. (**C**) SOD activation. (**D**) CAT activation. (**E**) ELISA detection for HO-1 protein expression. (**F**) MDA content. Experiments were carried out into control group, LPS infection group, Mel+LPS group and Mel group. (**G**) Western blotting using to detect p-Nrf2/Nrf2 expression. TM treated with LPS, Mel and/or SB203580. (**H**) ELISA used to detect HO-1 protein expression. TM treated with LPS, Mel, ML385 and/or SB203580. Data are shown as mean ± SEM. Different superscript letters (a–c) in each column representative statistically significant differences (*p* < 0.05).

The mechanisms by which melatonin reduces testicular oxidative stress due to LPS were also studied (Figure 5A). We observed that LPS administration inhibited SOD, GSH and CAT activities. Unexpectedly, melatonin did not significantly augment SOD, GSH and CAT activation in the LPS-treated group (*p* > 0.05) (Figure 5B–D); however, the level of HO-1 protein increased significantly (*p* < 0.05) (Figure 5E). Thus, melatonin attenuates oxidative damage of testicular macrophages by scavenging free radicals through up-regulating HO-1 translation (Figure 5F). Results showed that melatonin increased Nrf2 expression, while the p38MAPK inhibitor, SB203580, reduced phosphorylation Nrf2 (*p* < 0.05) (Figure 5G). HO-1 expression was also decreased significantly by SB203580 administration (*p* < 0.05) (Figure 5H). The findings indicate that melatonin regulates Nrf2 expression by inhibiting p38MAPK expression which, in turn, influences HO-1 expression in TM.

### 3.6. Melatonin Increases Testosterone Levels in Leydig Cells Co-Cultured with Testicular Macrophages

**Figure 6:**
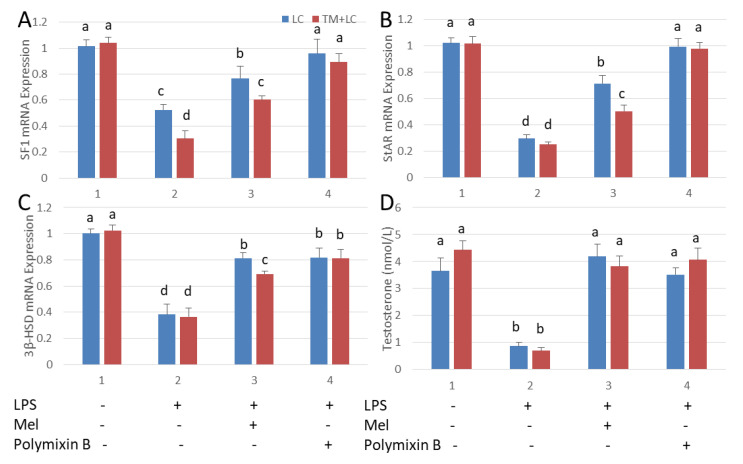
Melatonin increases testosterone content in an LPS-induced Leydig cell co-cultured system with testicular macrophages. qRT-PCR detection (**A**) SF1, (B) StAR, (**C**) mRNA expression and (**D**) testosterone levels in Leydig cell (LC) and in co-cultures with testicular macrophages (TM) detected by ELISA. Cell treated with LPS, Mel and/or polymixin B. Data are shown as mean ± SEM. Different superscript letters (a–d) in each column representative statistically significant differences (*p* < 0.05).

Leydig cells were co-cultured with testicular macrophages. As a result of LPS stimulation, mRNA expressions of SF1, StAR and 3β-HSD and plasma testosterone concentration were significantly depressed relative to those in the untreated control tissue (*p* < 0.05). With melatonin treatment, transcription of SF1, StAR and 3β-HSD and the level of testosterone were increased. Similar results were observed after treatment with polymixin B, a TLR4 inhibitor (Figure 6). The mRNA expressions of SF1, StAR and 3β-HSD in the co-cultured tissues were significantly lower than that in Leydig cells cultured alone (*p* < 0.05). This indicates that cytokines secreted by testicular macrophages influenced the expression of Leydig cell steroid synthesis-related genes.

### 3.7. Melatonin Alleviates LPS-Induced Orchitis in Sheep

**Figure 7:**
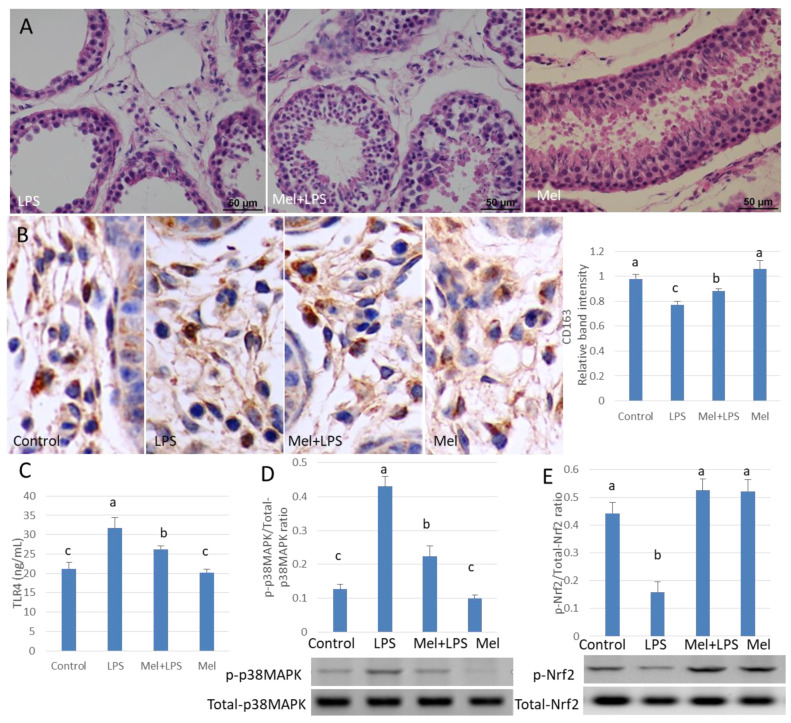
Melatonin alleviates LPS-induced sheep orchitis. (**A**) Testis histopathological HE stains. (**B**) Expression of immunohistochemical CD163 in testicular tissue. (**C**) ELISA detects TLR4 protein expression. Protein expression of (**D**) p-p38MAPK/p38MAPK and (**E**) p-Nrf2/Nrf2 was detected by Western blotting. All the above experiments were divided into a control group (testicular local injection of saline), LPS injection group, LPS + Mel injection testis group and Mel injection testis group. Data are shown as mean ± SEM. Different superscript letters (a–c) in each column representative statistically significant differences (*p* < 0.05).

**Figure 8:**
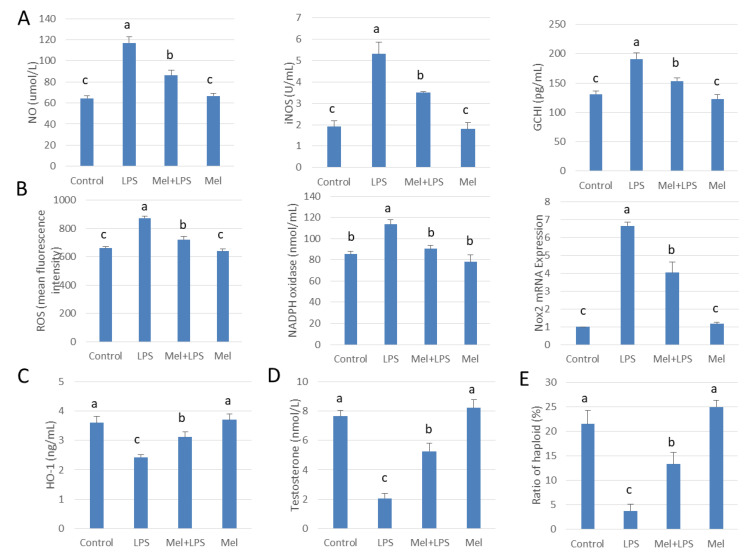
Melatonin administration increases haploid spermatogenic cell content in LPS-induced sheep orchitis. (**A**) NO content, iNOS viability and GCH1 protein expression. (**B**) ROS content, NADPH oxidase vitality and mRNA expression of Nox2. ELISA detected (**C**) HO-1 versus (**D**) testosterone content. (**E**) Percentage of haploid spermatogenic cells flow analysis. All the above experiments were divided into a control group (testicular local injection of saline), LPS injection group, LPS+Mel injection testis group and Mel injection testis group. Data are shown as mean ±SEM. Different superscript letters (a–c) in each column representative statistically significant differences (*p* < 0.05).

HE staining showed that the spermatogenic cells were preserved in seminiferous tubule after treatment with melatonin (Figure 7A). Immunohistochemistry was used to observe the expression of macrophage CD163 among macrophages. CD163+ expression cells were significantly increased in melatonin-treated tissues, compared to that in the LPS-treated tissues (*p* < 0.05) (Figure 7B). Also, in LPS-injected testis melatonin increased p-Nrf2 and HO-1, and reduced TLR4, p-p38MAPK translation levels. NO and ROS levels were also found to be diminished by melatonin treatment (Figure 7C–E and Figure 8A–C). In LPS-injected tissues, melatonin administration increased testosterone content and haploid sperm cell numbers in the seminiferous tubules (Figure 8D,E). Collectively, the data show that melatonin targets testicular tissue and activates local pro-phagocytic activity, decreases TLR4-mediated inflammatory factor expression and reduces oxidative stress.

## 4. Discussions

Although the testis is known as an immune privileged site a variety of bacteria and viruses still infect testes and induce orchitis [24]. Notably, most of the viruses that cause orchitis are RNA viruses; DNA virus infection rarely causes orchitis. Studies have shown that injection of LPS from *E. coli* causes orchitis, and inhibits mouse testosterone synthesis and spermatogenesis [25]. In the testicular environment, macrophages adopt an alternatively activated phenotype, including the decrease in proinflammatory gene expression, and constitutively produce IL-10 [26]. TMs are important contributors to the response of the testis to infection. In the early stage of testicular infection, macrophages secrete a large number of pro-inflammatory cytokines that contribute to the pathological process. In testis of infertile animals, the number of HLA-DR (+) macrophages was increased. The antigen-presenting ability of HLA-DR (+) macrophages was elevated and their ability to secrete IL-1, IL-6, TNF-α and NO was likewise enhanced [27]. This led to inhibition of testosterone secretion from Leydig cells. Unlike peritoneal macrophages, LPS-stimulated TM lack pro-inflammatory cytokine secretion and are refractory to NF-κB activation [28]. Melatonin reduces both acute and chronic inflammation [29,30]. There was a negative correlation between testicular melatonin concentration and the number of macrophages in the biopsy of infertile animals [31]. Melatonin inhibited the expression of pro-inflammatory cytokines TNF-α and IL-1β, and the expression of COX-2 in testicular macrophages [32]. Melatonin blocks the polarization of macrophages to the pro-inflammatory M1 phenotype [33]. Our results showed that melatonin increased the number of CD163+ testicular macrophages, promoted the phagocytic ability of testicular macrophages, and improved the level of anti-inflammatory IL-10. Likewise, melatonin attenuates the expression of MCP-1, TNF-α, IL-6 and IL-1βin secretory macrophages to relieve LPS-induced testicular inflammation.

Melatonin regulates the transcriptional activity of NF-κB and MAPKs [34]. Prophylactic treatment of melatonin is via TLR4 inhibiting pro-inflammatory cytokine production [35]. LPS stimulation of RAW264.7 could lead to NF-кB over-activation. After the addition of melatonin, the translocation of NF-κB subunit p65 and p50 significantly decreased, and reduced the transcriptions of downstream genes [36]. Melatonin exerts a protective effect in the intestine during secondary IAH primarily by attenuating the inflammatory responses which is in part attributable to p38MAPK inhibition [37]. Melatonin may play an important anti-inflammatory role by controlling macrophage polarization. The specific molecular mechanism of melatonin in controlling the balance between pro-inflammatory M1 macrophages and anti-inflammatory M2 macrophages remain to be determined [38]. We hypothesized that melatonin may convert the pro-inflammatory M1 macrophage phenotype to the anti-inflammatory M2 macrophage by inhibiting the p38MAPK signaling pathway and decreasing the translocation of NF-κB in LPS-induced sheep testicular macrophages. In an LPS-stimulated cultured human vascular smooth muscle cell line, CRL1999, p38MAPK phosphorylation and IκB-α phosphorylation were reduced and the binding of NF-кB and its promoter were inhibited [39]. p38MAPK plays an important role in the activation of NF-κB signaling pathways [40]. TM maintains testicular immune privilege by inhibiting NF-κB signal transduction and decreasing TLR cascade gene expression. Compared with peritoneal macrophages, TM has a lower constitutive expression of TLR pathway-specific genes. Moreover, the NF-κB signaling pathway is blocked in TM stimulated with LPS. Instead, challenging TM with LPS induces MAPK signaling pathways, which leads to the production of pro-inflammatory cytokines [41]. Thus, LPS-stimulated TM promotes the MAPK signaling pathway to produce pro-inflammatory cytokines. Our results showed that, melatonin inhibits p38MAPK activation in LPS-infected sheep testis and testicular macrophages. NF-κB activation was inhibited by adding p38MAPK inhibitor SB203580, suggesting that melatonin regulates NF-κB activation in sheep testicular macrophages by inhibiting p38MAPK phosphorylation.

Excessive oxidative stress induces apoptosis in germ cells. Melatonin efficiently scavenges free radicals produced by macrophages. Melatonin limits peritoneal macrophage NO and ROS production by inhibiting p38MAPK pathway [42]. The activity of NADPH oxidase leads to the up-regulation of gp91phox through the NF-𝜅B pathway. The inhibition of Nox2 expression by melatonin may be an important factor in its antioxidant effect [43]. Activation of p38MAPK triggers Nox2 expression and promotes ROS production [44]. In addition, the TLR4-Nox2 axis also modulates the phagocytic activity of macrophages [45]. Our results showed that melatonin regulates Nox2 expression, which depresses NADPH oxidase activity, and reduces ROS production in LPS-induced sheep orchitis. 

BH4 deficiency results in a decrease of iNOS uncoupling. The rate limiting enzyme of BH4 is GTP cyclohydrolase, which is encoded by GCHI. GCHI plays an important role in iNOS activation and is closely related to various pathological processes [46]. The anti-inflammatory effect of melatonin in glial cells after stimulation with pro-inflammatory cytokines is attributed to p38 inhibition, which down regulates the expression of iNOS [47]. Our results show that melatonin may inhibit iNOS activity through p38MAPK/GCH1 pathways, reducing NO production in LPS-induced sheep orchitis. 

Enzymes such as SOD, GSH, CAT and HO-1 are the main antioxidant enzymes in the male reproductive system. Studies have shown that melatonin protects the liver from I/R injury by HO-1 overexpression, which suppresses the TLR4 signaling pathway [48]. p38MAPK/Nrf2/HO-1 signaling is an important anti-inflammatory route in macrophages [49]. Increased HO-1 activity induced by p38MAPK activation has been shown to inhibit HMGB1 release under septic conditions both in vitro and in vivo [50]. Our results show that LPS inhibited SOD, GSH and CAT activity in testicular macrophages. Melatonin up-regulates HO-1 expression through p38MAPK/Nrf2 pathways to relieve oxidative damage in sheep testicular inflammation.

## 5. Conclusions

**Figure 9:**
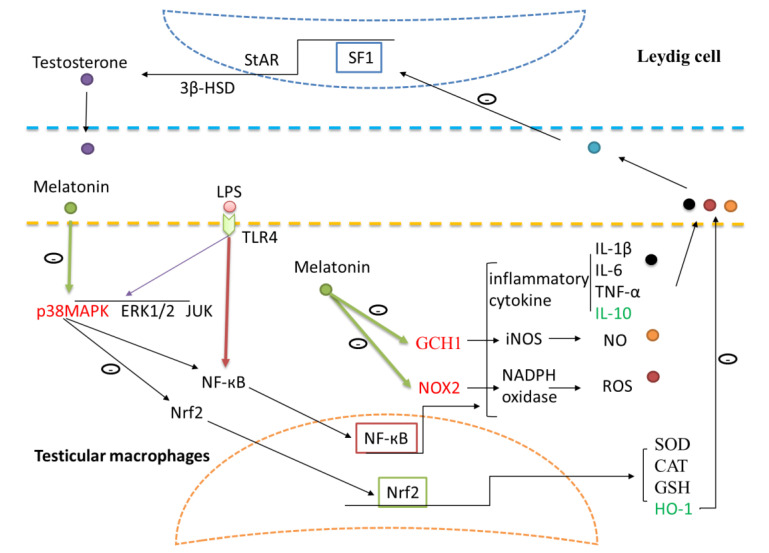
Schematic illustration of the proposed pathways by which melatonin regulates testosterone production and protects the testicular tissue from oxidative damage in LPS-induced sheep orchitis. Melatonin inhibited NF-кB through p38MAPK pathways in testicular macrophages. Expressions of GCHI and Nox2 were reduced by melatonin while HO-1 expression was up-regulated. Toll like receptors 4 (TLR4), nuclear factor kappa B (NF-кB), superoxide dismutase (SOD), catalase (CAT), glutathione (GSH), interleukin (IL), tumor necrosis factor alpha (TNF-α), heme oxygenase-1 (HO-1), 3β-hydroxysteroid dehydrogenase (3β-HSD), steroidogenic factor 1 (SF1), steroidogenic acute regulatory protein (StAR), nitric oxide (NO), Guanosine triphosphate cyclohydrolase I (GCHI), nitric oxide synthetase (iNOS), Reactive oxygen species (ROS), nicotinamide adenine dinucleotide phosphate (NADPH) oxidase, NADPH oxidase 2 (Nox2).

During inflammation, chemical agents and free radicals secreted by testicular macrophages inhibit the expression of steroid synthesis related proteins and genes in Leydig cells; this is associated with down-regulation of testosterone synthesis [51]. Most often the primary cause of infertility in human males is inflammation or autoimmunity. Testicular inflammation resulting from bacterial infections of the genitourinary system, sexually transmitted diseases, and non-reproductive system infections (e.g., mumps, SARS, and tuberculosis) often leads to temporary or permanent male infertility. Our results show that by activating TLR4 signaling pathways, melatonin inhibited transcription of regulatory factor NF-кB through p38MAPK pathways in testicular macrophages. As a result, IL-10 levels were elevated to resist the inflammatory response. Also, the synthesis of testosterone in testicular Leydig cells was promoted. Melatonin decreased expression of GCH1 and Nox2 in testicular macrophages via the TLR4 pathway. These changes then reduced iNOS and NADPH oxidase activity, removed the excessive free radical-mediated oxidative damage and restored testosterone synthesis in Leydig cells. Due to the participation of the Nrf2 pathway, melatonin upregulates HO-1 antioxidant protein expression to ease the level of oxidative stress in orchitis (Figure 9). This study provides a reference for melatonin treatment of orchitis caused by Gram-negative bacteria, and also suggests the use of methoxyindole as a treatment of orchitis caused by RNA virus infection.
